# 8-Methoxypsoralen has Anti-inflammatory and Antioxidant Roles in Osteoarthritis Through SIRT1/NF-κB Pathway

**DOI:** 10.3389/fphar.2021.692424

**Published:** 2021-09-06

**Authors:** Jichao Li, Zeng Zhang, Jinan Qiu, Xiaohan Huang

**Affiliations:** ^1^ The Third Department of Knee Injury, Luoyang Orthopedic Hospital of Henan Province, Orthopedic Hospital of Henan Province, Luoyang, China; ^2^ Zhengzhou Orthopedic Hospital, Zhengzhou, China

**Keywords:** osteoarthritis, 8-methoxypsoralen, inflammation, oxidative stress, SIRT1

## Abstract

Osteoarthritis (OA) is mainly manifested by joint pain, stiffness and mobility disorder, which is the main cause of pain and disability in middle-aged and elderly people. In this study, we aimed to explore the role and mechanism of 8-Methoxypsoralen (8-MOP) in the OA model both *in vitro* and *in vivo*. The rat chondrocytes were treated with IL-1β, and the proliferation, apoptosis, inflammatory reactions and oxidative stress responses were determined after treatment with different concentrations of 8-MOP. Real-time quantitative polymerase chain reaction (qRT-PCR) and/or Western blot were implemented to check the AMPK/SIRT1/NF-κB expression in chondrocytes. The NF-κB activity was determined by dual luciferase experiment. The pain threshold of OA rat model dealt with 8-MOP and/or the SIRT1 inhibitor EX527 was measured. Our results revealed that 8-MOP evidently reduced IL-1β-mediated apoptosis and inhibition of proliferation, and mitigated the expression of inflammatory cytokines and oxidative stress factors in chondrocytes. Additionally, 8-MOP promoted phosphorylated level of AMPKα, enhanced SIRT1 expression and inhibited the phosphorylation of NF-κB. After treatment with EX527, 8-MOP-mediated protective effects on chondrocytes were mostly reversed. *In vivo*, 8-MOP obviously improved the pain threshold in the OA rat model and reduced the injury and apoptosis of chondrocytes in the joints. In addition, 8-MOP relieved inflammatory and oxidative stress responses in the articular cartilage via enhancing SIRT1 and repressing NF-κB activation. After the treatment with EX527, the 8-MOP-mediated protective effects were distinctly weakened. In summary, our study testified that 8-MOP alleviates pain, inflammatory and oxidative stress responses in OA rats through the SIRT1/NF-κB pathway, which is expected to become a new reagent for clinical treatment of OA.

## Introduction

Osteoarthritis (OA) is a prevalent form of chronic joint inflammation, and the incidence of which may increase with the extension of human life expectancy (Pereira et al., 2015). Researches have exhibited that the occurrence of OA is related to genetic, biological and biomechanical factors (Glyn-Jones et al., 2015). OA is a degenerative joint disease involving cartilages and their surrounding tissues, which progresses slowly and is often accompanied by pain and limited joint movement (Litwic et al., 2013). Thus, OA not only lowers the patients’ quality of life but also increases certain social and economic costs. Current therapies for OA are mainly related to intraarticular, physical, alternative, and surgical therapy (Abramoff and Caldera, 2020). Many agents have been found to be effective in relieving the pain in OA patients, those drugs include analgesics such as non-steroidal anti-inflammatory drugs, corticosteroids such as glucocorticoids, hyaluronic acid and local anesthetics (Rychel, 2010; Richards et al., 2016; Jones et al., 2019). However, the limitations such as side effects have restrained their clinical application. Therefore, it is indispensable to explore new therapeutic agents for the treatment of OA.

OA was once defined as a non-inflammatory form of arthritis, but now OA is considered to be characteristic with overproduced inflammatory response (Shen et al., 2017). Notably, oxidative stress contributes to the pathogenesis of OA (Zhuang et al., 2018). Multiple drugs exert therapeutic effects on OA through the anti-inflammatory and antioxidant effects. For instance, quercetin reduces oxidative stress-induced chondrocytes apoptosis by regulating the SIRT1/AMPK pathway and prevents OA evolvement in mice (Feng et al., 2019a). Moreover, researches have revealed that aucubin can slow OA progression in mice by suppressing chondrocyte apoptosis and reactive oxygen species (ROS) production (Wang et al., 2019). Both quercetin and aucubin are natural compounds with anti-inflammatory and antioxidant properties. Therefore, we hypothesized that the drugs with anti-inflammatory or antioxidant properties could serve as potential agents for OA treatment.

8-Methoxypsoralen (8-MOP) is a natural furanocoumarin with various biological activities (Liu et al., 2017). 8-MOP is a photosensitizer, and it has been applied to immunotherapy (Hähnel et al., 2020). 8-MOP has been found with multiple biological function, such as regulation of apoptosis and proliferation in tumors. One study has shown that 8-MOP inactivates the PI3K/Akt pathway and induces apoptosis, thereby inhibiting the growth of neuroblastoma and colon cancer cells (Bartnik et al., 2017). What’s more, 8-MOP dampens the proliferation of DU145 prostate cancer cells and facilitates cell apoptosis (Sturaro et al., 2017). Notably, some scholars identified that 8-MOP isolated from plants hampers the release of interleukin (IL)-6 and tumor necrosis factor (TNF)-α in RAW 264.7 cells by inhibiting the IKK/IκB/NF-κB pathway (Yang et al., 2015). These findings suggest that 8-MOP has distinct anti-inflammatory and antioxidant effects. However, the effects of 8-MOP on OA remain unclear.

Sirtuin-1 (SIRT1) is a class III histone deacetylase (HDAC) and an NAD+ dependent enzyme that is widely involved in gene regulation, cell growth and development (Meng et al., 2017; Alves-Fernandes and Jasiulionis, 2019). Studies have shown that SIRT1 is a target for the treatment of diabetic complications (Strycharz et al., 2018). Additionally, SIRT1 improves fatty liver-mediated hepatitis by regulating liver lipid metabolism and controlling liver oxidative stress (Ding et al., 2017). This suggests that SIRT1 plays a vital role in metabolic diseases. Other studies have revealed that Pinolenic acid suppresses lipid accumulation, oxidative stress and oleic acid-induced inflammation in HepG2 cells through the AMPK/SIRT1 pathway (Zhang et al., 2019), indicating that SIRT1 may be involved in modulating cellular inflammation and oxidative stress. On the other hand, the NF-κB pathway has been considered as a classical pro-inflammatory signaling pathway (Lawrence, 2009), which is closely coordinated with other signaling pathways to modulate the biological behaviors of cells (Oeckinghaus et al., 2011). Additionally, the NF-κB signaling has been identified to aggravate OA by inducing cartilage matrix remodeling, chondrocyte apoptosis, synovitis inflammation, and so on (Lepetsos et al., 2019). Suppressing NF-κB pathway activation is effective in treating OA. For example, Cortistatin alleviates OA *in vivo* by inhibiting the activation of the NF-κB pathway (Zhao et al., 2019). However, it is not clear whether the effect of 8-MOP on OA is related to SIRT1 and the NF-κB pathway.

In summary, our study aims to explore the role and mechanism of 8-MOP on the inflammatory and oxidative stress responses of OA on both *in-vivo* and *in-vitro* OA models. Our results exhibited that 8-MOP reduced inflammatory and oxidative stress responses in OA by upregulating SIRT1 and inhibiting the NF-κB pathway, suggesting that 8-MOP is a promising agent in relieving OA progression.

## Materials and Methods

### Cell Culture

Rat chondrocytes were purchased from American Type Culture Collection (ATCC, Rockville, MD, United States). The chondrocytes were cultured in the DMEM medium (Thermo Scientific Hyclone, UT, United States) supplemented with 10% FBS and 1% penicillin/streptomycin and incubated at 37°C with 5% CO_2_ and saturated humidity. The cell growth was observed regularly, and the culture medium was changed every 2–3 days. The cells in the logarithmic growth phase were utilized for subsequent experiments. An *in vitro* OA model was induced by treating chondrocytes with IL-1β (20 ng/ml) for 24 h.

### 3-(4,5-Dimethylthiazol-2-yl)-2,5-diphenyltetrazolium Bromide Assay

The chondrocytes were diluted into cell suspension (4 × 10^4^/ml) using a complete medium and seeded into 96-well culture plates at 200 μl/well. After the cells adhered to the wall, 0.5% FBS medium was utilized to synchronize the cells, and the culture medium was absorbed and discarded after 24 h 200 μl of corresponding culture solution with drug-containing serum was added to each well, with three wells for each group. After 24, 48, and 72 h of culture, the culture medium was removed, and 190 μl culture medium plus 10 μl of MTT solution (5 mg/ml) (MedChem Express, NJ, United States) was added into each well, and the cells were incubated in a 37°C incubator with 5% CO_2_ and saturated humidity for 4 h. After that, the incubation solution was discarded, and 150 μl of dimethyl sulfoxide (DMSO, Solarbio, Beijing, China) was added to each well. Then, the cells were oscillated and incubated for 10 min. After the precipitation was completely dissolved, the optical density (OD) value was measured at the wavelength of 570 nm on the Multiskan Spectrum microplate reader (Thermo Scientific, Logan, UT, United States).

### Flow Cytometry

According to the instructions of the manufacture, the AnnexinV/7-AADA apoptosis Detection Kit (Southern Biotechnology, Birmingham, Al, United States) was used for evaluating the apoptosis of chondrocytes. After the treatment with IL-1β and/or 8-MOP, EDTA-free trypsin (0.25%, Beyotime, Shanghai, China) was used for the digestion of chondrocytes, which were then centrifuged at 2000 rpm for 5 min, and then the medium was discarded. After being rinsed with cold PBS, the cells were centrifuged at 2000 rpm for 5 min, and the supernatant was discarded. One sample was reserved without adding 7-AAD. We added 5 μl of 7-AAD staining solution to 50 μl BindingBuffer and mixed them well, so as to suspend the cells. Cells in each group were added to 450 μl BindingBuffer and mixed well gently. Subsequently, cells in each group were supplemented with 1 μl AnnexinV-PE and reacted at room temperature (RT) in the dark for 15 min. One sample was retained without adding AnnexinV-PE. FCM was performed immediately on NovoCyte Flow Cytometer (ACEA Biosciences, China), and the results were presented as apoptosis rate. Apoptosis rate = apoptotic cell number/(apoptotic cell number + normal cell number) ×100%.

### Western Blot

Chondrocytes treated with IL-1β and/or 8-MOP were taken. The rat cartilage was treated with a homogenizer and centrifuged, and then the supernatant was discarded. RIPA lysate (Beyotime Biotechnology, Shanghai, China) was utilized to extract the total protein of the cells and tissues in each group. 50 μg of total protein was loaded on 12% polyacrylamide gel for electrophoresis at 100 V for 2 h and then electrotransferred to polyvinylidene fluoride (PVDF) membranes (Sigma Aldrich, China, Shanghai). After being blocked with 5% skimmed milk at RT for 1 h, the membranes were rinsed with TBST 3 times for 10 min each time. Then, the following primary antibodies were added and incubated overnight at 4°C: Bax (1:2000, ab182733), Bcl2 (1:2000, ab182858), Caspase3 (1:2000, ab184787), SIRT1 (1:1,000, ab189494), NF-κB p65 (1:2000,ab32536), p-NF-κB p65 (1:2000, ab76302), NF-κB p65 (Acetyl K310) (1:1,000, ab19870), *p*-AMPKα (phospho S496) (1:1,500, ab92701) and β-actin (1:2000, ab8227). After being washed with TBST, the membranes were incubated with the Goat-anti Rabbit IgG (1:2000, ab6721) for 1 h at RT. Subsequently, the membranes were rewashed with TBST 3 times. Finally, the ECL kit (Amersham Pharmacia Biotech, Little Chalfont, United Kingdom) was applied for color imaging, and Image J was taken to analyze the gray intensity of each protein. β-actin was used as internal control of the target proteins. All the antibodies were purchased from Abcam company (MA, United States).

### Enzyme-Linked Immunosorbent Assay

The chondrocytes treated with IL-1β and/or 8-MOP for 24 h were seeded into 6-well plates, with four duplicate wells in each group. After 48 h of further culture, the cell supernatant was collected and centrifuged at 1,000 rpm at 4°C for 10 min. Then, the supernatant was taken again. The cartilage tissues of rats in each group were homogenized with the lysate (Beyotime, Shanghai, China) containing 1% protease inhibitor Cocktail (Roche, Basel, Switzerland), centrifuged at 14,000 rpm at 4°C for 25 min, and the supernatant was retained. The contents of inflammatory cytokines (IL-6, TNF-α, and IL-18) in the supernatant were respectively tested by ELISA kits (Invitrogen, CA, United States). All the experimental procedures were performed strictly in accordance with the kit requirements.

### Oxidative Stress Factors Detection

The oxidative stress factors (SOD, GSH-PX, and MDA) in chondrocytes and tissues were checked by the detection kits purchased from Nanjing Jiancheng Bioengineering Institute (Nanjing, China). Those detection kits included SOD (cat.no.A001-3-2), GSH-PX (cat.no.A005-1-2) and MDA (cat.no.A003-1-2). All the experimental procedures were performed strictly in accordance with the kit requirements.

### Real-Time Quantitative Polymerase Chain Reaction

In this experiment, Primer3.0 software was applied to design the primers of the target gene, which were synthesized by Shanghai Sangon Biotechnology Service Co., Ltd. After the treatment of different factors, the total RNA was extracted from the chondrocytes and cartilage tissues of each group using the TRIzol reagent (Invitrogen, Carlsbad, CA, United States). The ultraviolet spectrophotometer (Raylabel Instrument Co., Ltd., Shanghai, China) was applied to test the purity and concentration of the RNA, which was then reversely transcribed into the first strand of cDNA. With this chain as the template, the mRNA fragments of IL-18, IL-6, TNF-α and SIRT1 were amplified by the ABI7300 quantitative fluorescence PCR instrument (Thermo Scientific Hyclone, UT, United States). GAPDH served as the internal reference, and the relative expression of the target genes was calculated by the formula RQ = 2^−∆∆CT^ Table 1.

**TABLE 1:** The primers of genes

Gene (mouse)	Primer sequences (5′-3′)
SIRT1	F: CAT​CGC​AGT​CTC​CAA​GAA​GC
	R: TGC​CAT​CAT​GAA​GCC​AGA​GA
IL-18	F: TGA​TAT​CGA​CCG​AAC​AGC​CA
	R: CCT​GGC​ACA​CGT​TTC​TGA​AA
IL-6	F: CCA​CTG​CCT​TCC​CTA​CTT​CA
	R: TTC​TGA​CAG​TGC​ATC​ATC​GC
TNF-α	F: ACC​AGG​AGA​AAG​TCA​GCC​TC
	R: GCT​GGG​TAG​AGA​ACG​GAT​GA
GAPDH	F: TGA​CTG​TGC​CGT​TGA​ACT​TG
	R: GAG​ACA​GCC​GCA​TCT​TCT​TG

### Rat Osteoarthritis model

In this experiment, thirty-six 5 week-old Wistar rats without underlying diseases were obtained from the Henan Experimental Animal Center. All rats were placed at preference temperature with 12 h of light/dark cycle and had free access to adequate food and water. All experimental procedures were ethically approved by Luoyang Orthopedic Hospital of Henan Province Institutional Animal Use and Care Committee [Approve number: SCXK (Yu) 2017-0001]. For the OA modeling, 0.4 ml of 4% papain solution was thoroughly mixed with 0.2 ml L-cysteine (0.03 mol L^−1^) and maintained for 30 min. Then, 30 μL of the mixture was taken and intraarticularly injected into the right knee of the rats, and the injection was repeated on the 5^th^ day. The rats’ survival status was closely monitored after the modeling, and the pain threshold of each group was examined on the 10^th^ day after the modeling. Six weeks later, all rats were killed using a rat euthanasia box filled with carbon dioxide (Shanghai Yuyan Scientific Instrument Co., Ltd.), and the synovium tissues of the right knee joint of the rats were cryopreserved for subsequent testing.

All the rats were randomly divided into six groups with six rats in each group: the sham group, the 8-MOP group, the OA group, the OA + EX527 group, the OA + 8-MOP group, and the OA + 8-MOP + EX527 group. The sham group: Only the same amount of sterile saline was injected. The 8-MOP group: 8-MOP (30 μmol/kg body weight) was injected. The OA + EX527 group: EX527 (20 μmol/kg body weight) were injected after modeling. The OA + 8-MOP group: 8-MOP (30 μmol/kg body weight) was injected after the modeling. The OA + 8-MOP + EX527 group: 8-MOP (30 μmol/kg body weight) and EX527 (20 μmol/kg body weight) were injected after modeling. All the above groups were administered by intraperitoneal injection, all doses were given once daily for nine consecutive days. The skin condition of rats was observed by naked eye. The blood and OA site of rats in the above groups were collected and analyzed at room temperature within 2 h after collection. The liver, kidney and knee tissues of rats in each group were collected and used for subsequent histopathological examination. The high-performance liquid chromatography-diode array detector (HPLC-DAD, Agilent1200, Agilent company, CA, United States) was used for determining the content of 8-MOP in the serum and OA site.

### Pain Scoring

The paw withdrawal threshold (PWT) was performed to evaluate the pain score of rats. On the 10^th^ day after the modeling, an electronic automatic claw tactile tester (Shanghai Huanxi Medical Instrument Co., Ltd.) was applied for the PWT measurement of mechanical stimulation in experimental rats (unit:g). In brief, the rats’ feet were stimulated with a stimulus needle, and the stimulation force was gradually facilitated, and the maximum force value during which the rats pulled the paw to remove the stimulation was recorded as PWT. The PWT was measured once every 10 min for five consecutive times, and the average value was taken.

### Haematoxylin-Eosin Staining

A HE staining kit (cat.no. C0105S, Beyotime, Shanghai, China) was used for histopathological examination of the liver, kidney knee tissues of rats. Frozen sections (4 μM) of liver and kidney tissue and cartilage tissues of the knee joint, were taken from rats. After rewarming and hydration, the sections were stained with hematoxylin dye solution. After differentiation with 1% hydrochloric acid ethanol, the sections were and washed with double distilled water. After staining with eosin dye solution, they were dehydrated with gradient alcohol, transparentized with xylene, mounted with resin, and observed under a microscope (Olympus, Tokyo, Japan).

### Safranin O/Fast Green Staining

The cartilage tissues of the rat knee joint were fixed with 4% paraformaldehyde and then decalcified with EDTA decalcifying solution (0.5 μM, pH = 8) for 15 days. The solution was changed every 3 days. Then, the tissues were embedded in paraffin and sliced into sections (4 μM thick). The pathological changes of the cartilage were observed by Safranin O/fast green staining (Sigma-Aldrich, St.Louis, MO, United States) in accordance with the manufacturer’s introduction.

### Immunohistochemistry

The rat cartilage tissue was fixed with 4% paraformaldehyde, dehydrated, transparentized, paraffin-impregnated, and embedded. After that, the coronal sections with a thickness of 5 μM were obtained. Then, the sections were repaired by microwave for 1 min, inactivated with H_2_O_2_ (3%) for 15 min, and blocked with BSA (5%) for 30 min. After spin-drying, the sections were incubated with the primary Caspase3 antibody (Abcam, 1:200, ab179517) overnight at 4°C. The next day, the Goat-anti rabbit IgG (Abcam, 1:1,000, ab6721) was added and incubated at RT for 30 min. Afterward, the sections were incubated with the DAB solution for 40 min and developed with DAB chromogen for 5 min. After the reaction, the sections were dehydrated, transparentized, mounted with neutral gum, and observed under the microscope.

### Dual Luciferase Experiment

The dual luciferase experiment was performed to test the NF-κB activity. The chondrocytes were collected and seeded in 24-well plates (1 × 105 cells/well). Then the cells were transfected with 500 ng of the NF-κB-dependent firefly luciferase (ELAM-luc), 500 ng of pRL-TK was used as internal control. 24 h after the transfection, the cells were washed with cold PBS for 2 times. The PLB lysate was added, and the Dual Luciferase Reporter System Kit (E1910, Promega, WI, United States) was used to detect firefly and renilla luciferase activity.

### Statistical Analysis

All experimental data in this study were analyzed with GraphPad Prism 8 (GraphPad Software, CA, United States). The measurement data were presented as x ± *s*. The *t* test was applied for comparison between two groups, and one-way ANOVA was applied for comparison between multiple groups. *p* < 0.05 was considered statistically significant.

## Results

### 8-MOP Attenuated IL-1β-Mediated Cell Apoptosis and Proliferation Inhibition

**FIGURE 1:**
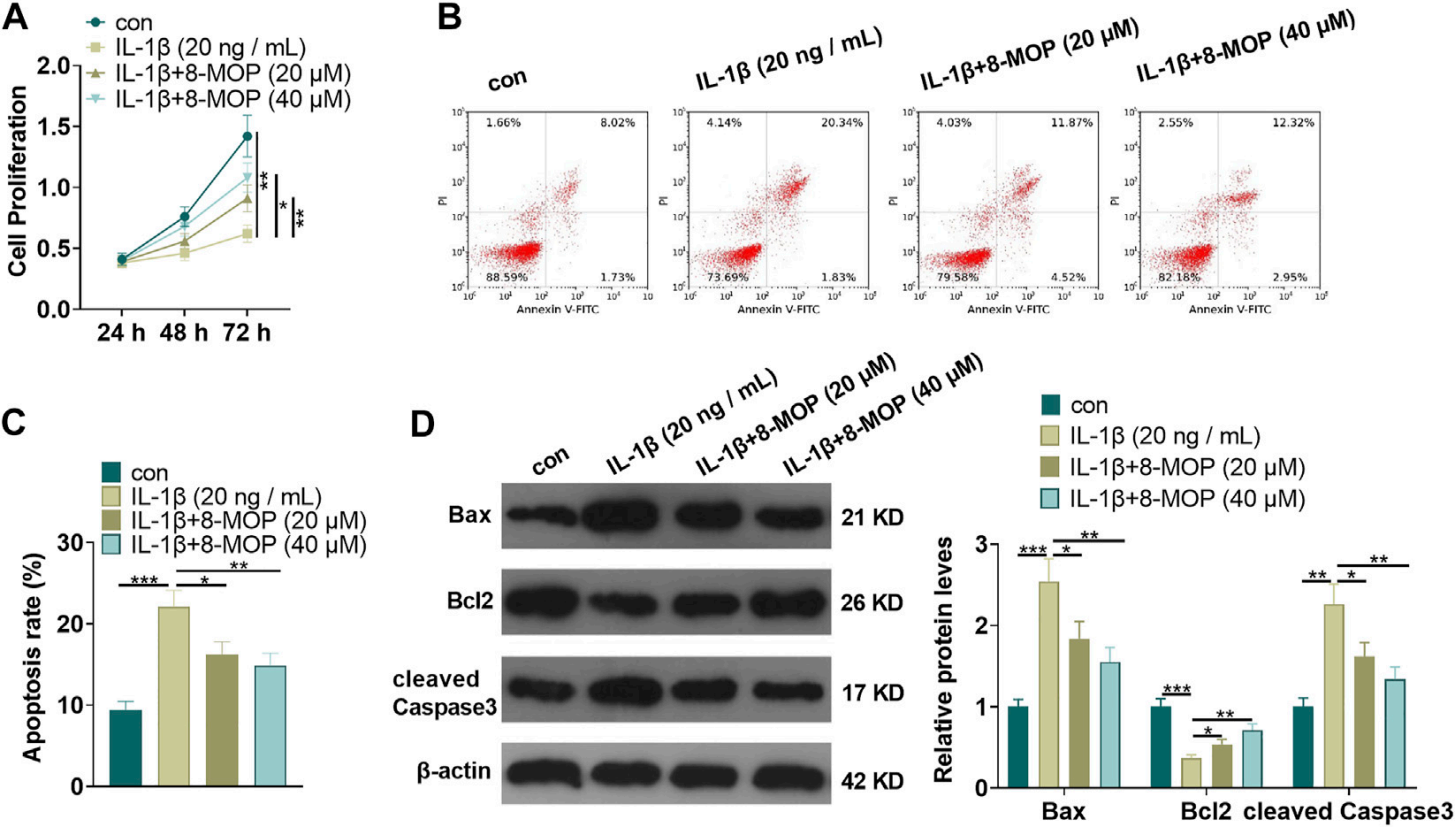
8-MOP attenuated IL-1β-mediated cell apoptosis and proliferation inhibition. Rat chondrocytes were treated with IL-1β (20 ng/ml) and/or 8-MOP (20 and 40 µM) for 24 h. **(A–C)**, MTT and FCM were applied to monitor cell proliferation and apoptosis, respectively. **(D)**, Western blot was utilized to measure the expression of apoptotic related proteins, including Bax, Bcl2, and cleaved Caspase3. *p < 0.05, **p < 0.01, ***p < 0.001. (*n* = 3).

On the basis of IL-1β treatment, rat chondrocytes were treated with 8-MOP (20, 40 μM). MTT and FCM were taken to define cell proliferation and apoptosis. As a result, compared with the con. group, cell proliferation was distinctly reduced and cell apoptosis was evidently enhanced after treatment with IL-1β. However, compared with the IL-1β group, 8-MOP obviously promoted the proliferation and inhibited apoptosis of chondrocytes (*p* < 0.05, Figures 1A–C). Further, Western blot was utilized to define the expression of apoptosis-related proteins. The results showed that IL-1β notably strengthened the expression of Bax and cleaved Caspase3 and inhibited the expression of Bcl2 in chondrocytes (vs. the con. group). In contrast, compared with the IL-1β group, the expression of Bax and cleaved Caspase3 was evidently dampened after the 8-MOP treatment, while the Bcl2 expression was elevated (*p* < 0.05, Figure 1D). All the results suggested that IL-1β induced apoptosis of rat chondrocytes and inhibited cell proliferation, while 8-MOP significantly reversed IL-1β-mediated effects on chondrocytes.

### 8-MOP Weakened IL-1β-Mediated Inflammatory and Oxidative Stress Responses

**FIGURE 2:**
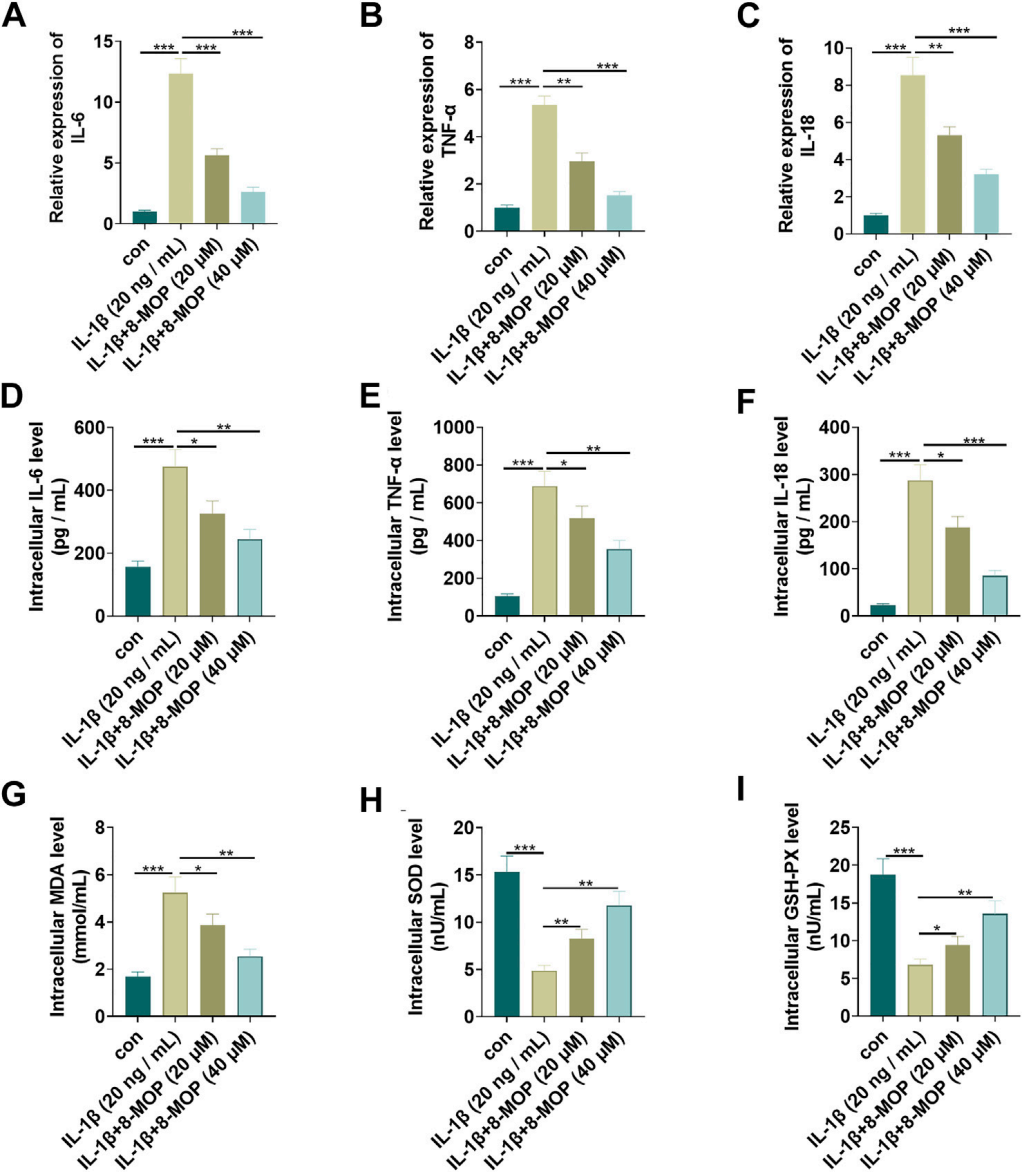
8-MOP weakened IL-1β-mediated inflammatory and oxidative stress responses. Rat chondrocytes were treated with IL-1β (20 ng/ml) and/or 8-MOP (20 and 40 µM) for 24 h. **(A–F)**. The levels of inflammatory cytokines (IL-6, TNF-α, and IL-18) were detected by qRT-PCR and ELISA. **(G–I)**: The oxidative stress factors (MDA, SOD, and GSH-PX) in chondrocytes were measured by the MDA, SOD, and GSH-PX detection kits. *p < 0.05, **p < 0.01, ***p < 0.001. (*n* = 3).

The rat chondrocytes were treated with IL-1β and/or 8-MOP (20, 40 μM) for 24 h. To understand the effects of IL-1β and 8-MOP on the inflammatory and oxidative stress responses of chondrocytes, we adopted qRT-PCR and ELISA to measure the release of inflammatory cytokines (including IL-6, TNF-α, and IL-18). The results exhibited that compared with the con. group, IL-1β distinctly enhanced the level of inflammatory cytokines (IL-6, TNF-α, and IL-18) both at the mRNA level and protein level (Figures 2A–F). Under the treatment of 8-MOP, IL-6, TNF-α, and IL-18 levels were all significantly with the increase of 8-MOP’s doses (Figures 2A–F). Additionally, the oxidative stress mediators, including MDA, SOD and GSH-PX in the culture medium were also evaluated. As the data showed, IL-1β enhanced MDA level while reduced SOD and GSH-PX levels in the culture medium. However, 8-MOP treatment reduced MDA and promoted SOD and GSH-PX levels (vs.IL-1β group, Figures 2G–I). These results indicated that 8-MOP weakened IL-1β-mediated inflammatory and oxidative stress responses.

### 8-MOP Enhanced SIRT1 and Suppressed the Phosphorylation of NF-κB

**FIGURE 3:**
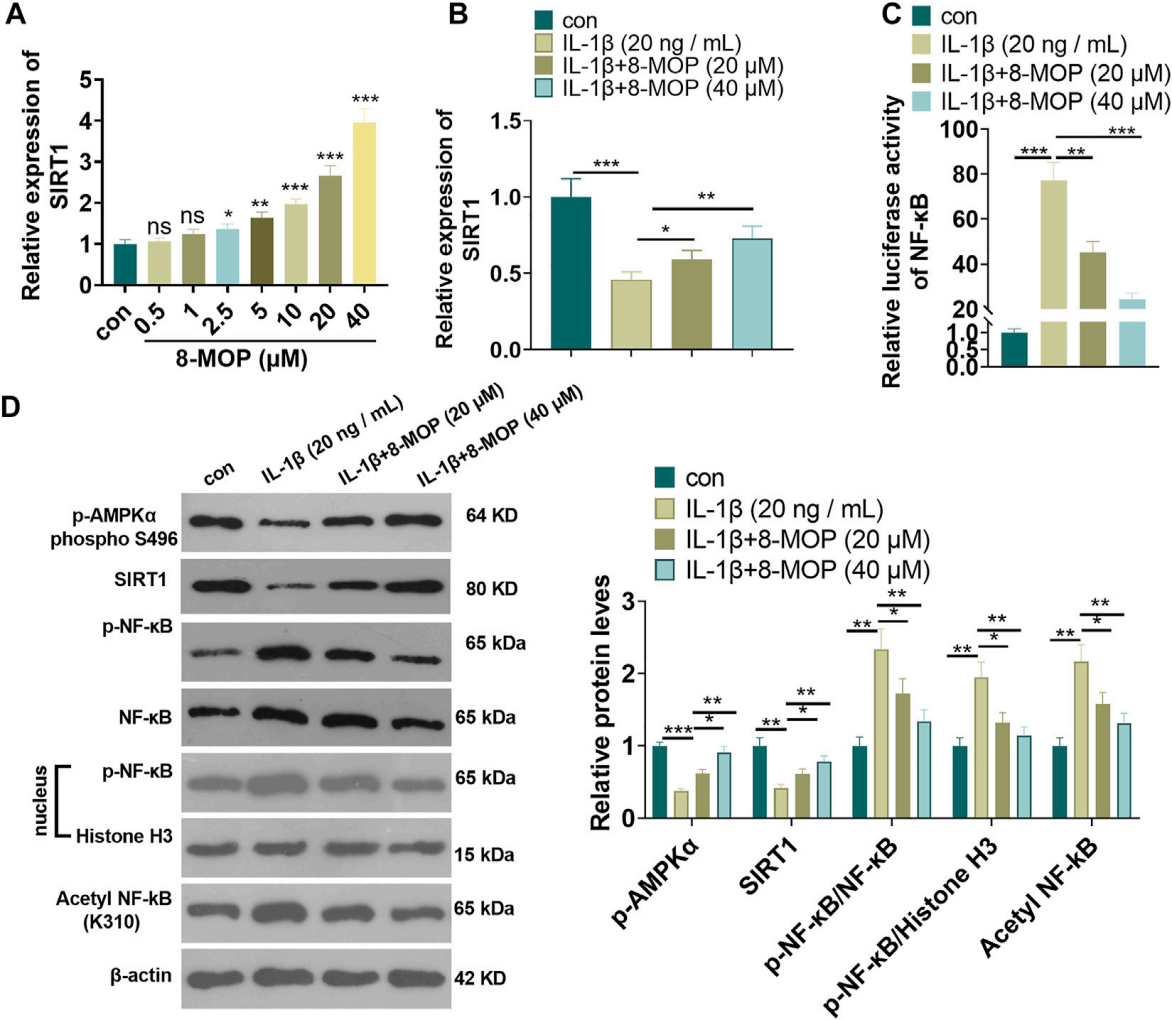
8-MOP enhanced SIRT1 and suppressed the phosphorylation of NF-κB. A. Different doses of 8-MOP (0, 0.5, 1, 2.5, 5, 10, 20, 40 µM) were used for treating chondrocytes for 24 h qRT-PCR was used for evaluating SIRT1 mRNA. Rat chondrocytes were treated with IL-1β (20 ng/ml) and/or 8-MOP (20 and 40 µM) for 24 h. **(B)**. qRT-PCR was used for evaluating SIRT1 mRNA. **(C)**. Dual luciferase experiment was used for evaluating the activity of NF-κB. **(D)**. The expression of p-AMPKα, SIRT1 and p-NF-κB p65 protein levels and acetylation NF-κB p65 was determined by Western blot, respectively. ns *p* > 0.05, *p < 0.05, **p < 0.01, ***p < 0.001. (*n* = 3).

To explore the underlying mechanism of 8-MOP in chondrocytes, we performed qRT-PCR to detect SIRT1 mRNA in chondrocytes treated 8-MOP (0, 0.5, 1, 2.5, 5, 10, 20, 40 μM). The data indicated that 8-MOP (2.5, 5, 10, 20, 40 µM) apparently enhanced the SIRT1 mRNA level (compared with con group, Figure 3A). Under the IL-1β, SIRT1 mRNA was inhibited, while 8-MOP significantly promoted SIRT1 mRNA level compared with the IL-1β group (Figure 3B). The activity of NF-κB was determined by luciferase experiment. The result showed that IL-1β enhanced NF-κB activity while 8-MOP treatment reduced its activity in a significant and dose-dependent manner (Figure 3C). What’s more, we performed western blot to detect the level of *p*-AMPKα, SIRT1 and NF-κB p65 in chondrocytes were treated with IL-1β and/or 8-MOP (20, 40 μM). As the data indicated, IL-1β dramatically inhibited *p*-AMPKα and SIRT1 levels, enhanced p-NF-κB p65 in the chondrocytes and nuclear, promoted acetylation NF-κB p65. However, after the treatment of 8-MOP, *p*-AMPKα and SIRT1 levels were both promoted, while p-NF-κB p65 in the chondrocytes and nuclear, and acetylation NF-κB p65 were all repressed by 8-MOP. The above results indicated that 8-MOP enhanced AMPK/SIRT1 and suppressed the phosphorylation of NF-κB.

### Inhibition of SIRT1 Weakened the Protective Effect of 8-MOP on Chondrocytes

**FIGURE 4:**
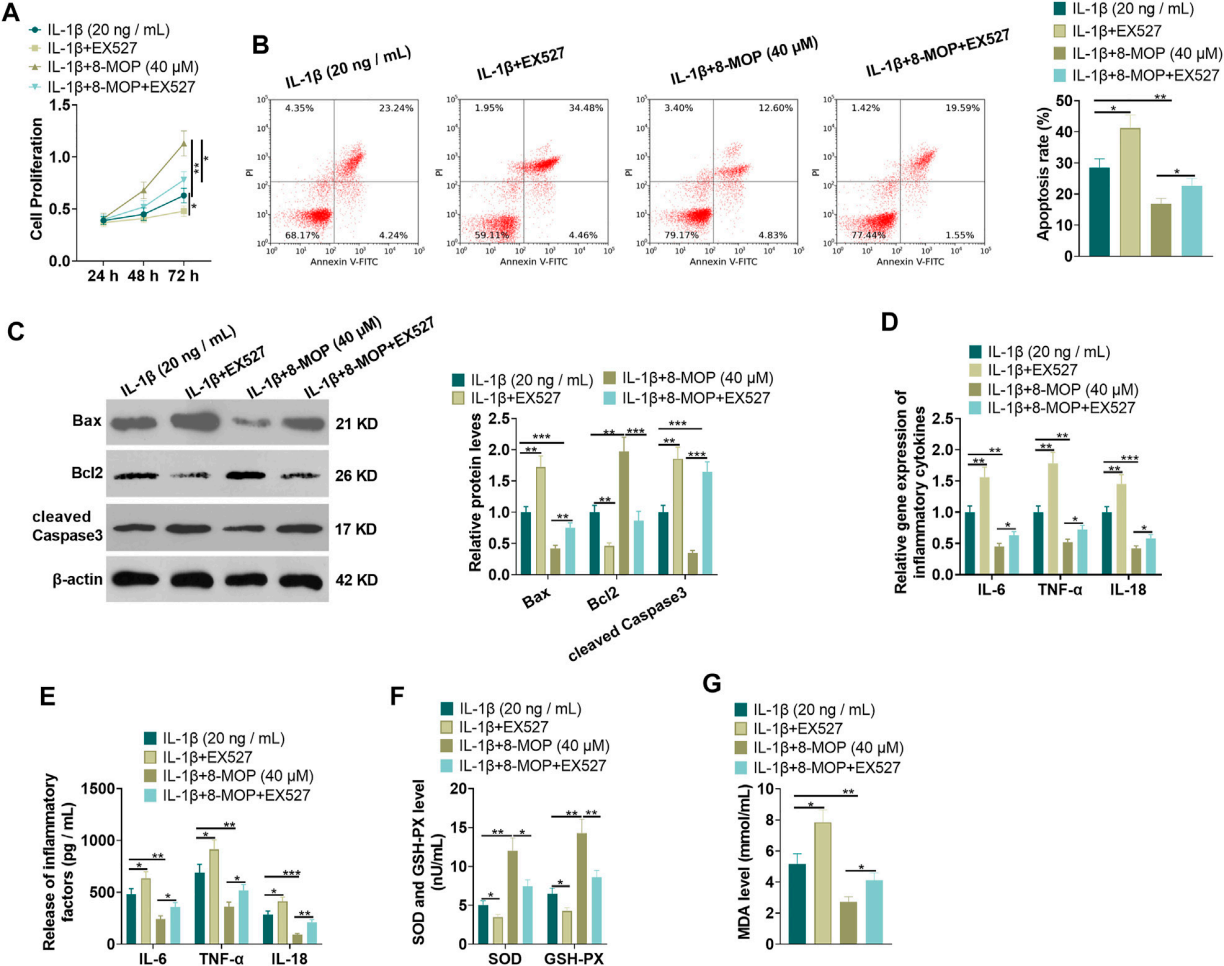
Inhibition of SIRT1 weakened the protective effect of 8-MOP on chondrocytes. On the basis of IL-1β stimulation, the chondrocytes were dealt with 8-MOP (40 μM) and/or EX527 (20 μM) for 24 h. **(A–B)**, MTT and FCM were adopted to define cell proliferation and apoptosis, respectively. **(C)**. The expression of Bax, Bcl2, and cleaved Caspase3 was checked by Western blot. **(D–E)**. The levels of inflammatory cytokines (IL-6, TNF-α, and IL-18) were detected by qRT-PCR and ELISA. **(F–G)**: The oxidative stress factors (MDA, SOD, and GSH-PX) in chondrocytes were measured by the MDA, SOD, and GSH-PX detection kits. *p < 0.05, **p < 0.01, ***p < 0.001. (*n* = 3).

To confirm the role of SIRT1 in 8-MOP-mediated effects, we administered chondrocytes with IL-1β, 8-MOP (40 μM) and EX527 (the SIRT1-specific inhibitor). MTT and FCM were performed for evaluating cell proliferation and apoptosis. The findings showed that compared with the IL-1β or IL-1β + 8-MOP (40 μM) group, EX527 distinctly inhibited chondrocyte proliferation and enhanced cell apoptosis (*p* < 0.05, Figures 4A,B). Western blot revealed that the expression of Bax and cleaved Caspase3 was evidently facilitated and the expression of Bcl2 was dampened after treatment with EX527 on the basis of the IL-1β or IL-1β + 8-MOP (40 μM) group (*p* < 0.05, Figure 4C). Next, the inflammatory cytokines and oxidative stress mediators were examined by qRT-PCR and ELISA. The results showed that compared with the IL-1β or IL-1β + 8-MOP (40 μM) group, EX527 enhanced the release of some cytokines (IL-6, TNF-α, IL-18) and oxidative stree mediator MDA, while the release of SOD and GSH-PX was reduced (*p* < 0.05, Figures 4D–G). The above experimental results indicated that inhibiting SIRT1 weakened the protective effect of 8-MOP on chondrocytes.

### 8-MOP Improved the Pain and Articular Cartilage Injury in Osteoarthritis Rats

**FIGURE 5:**
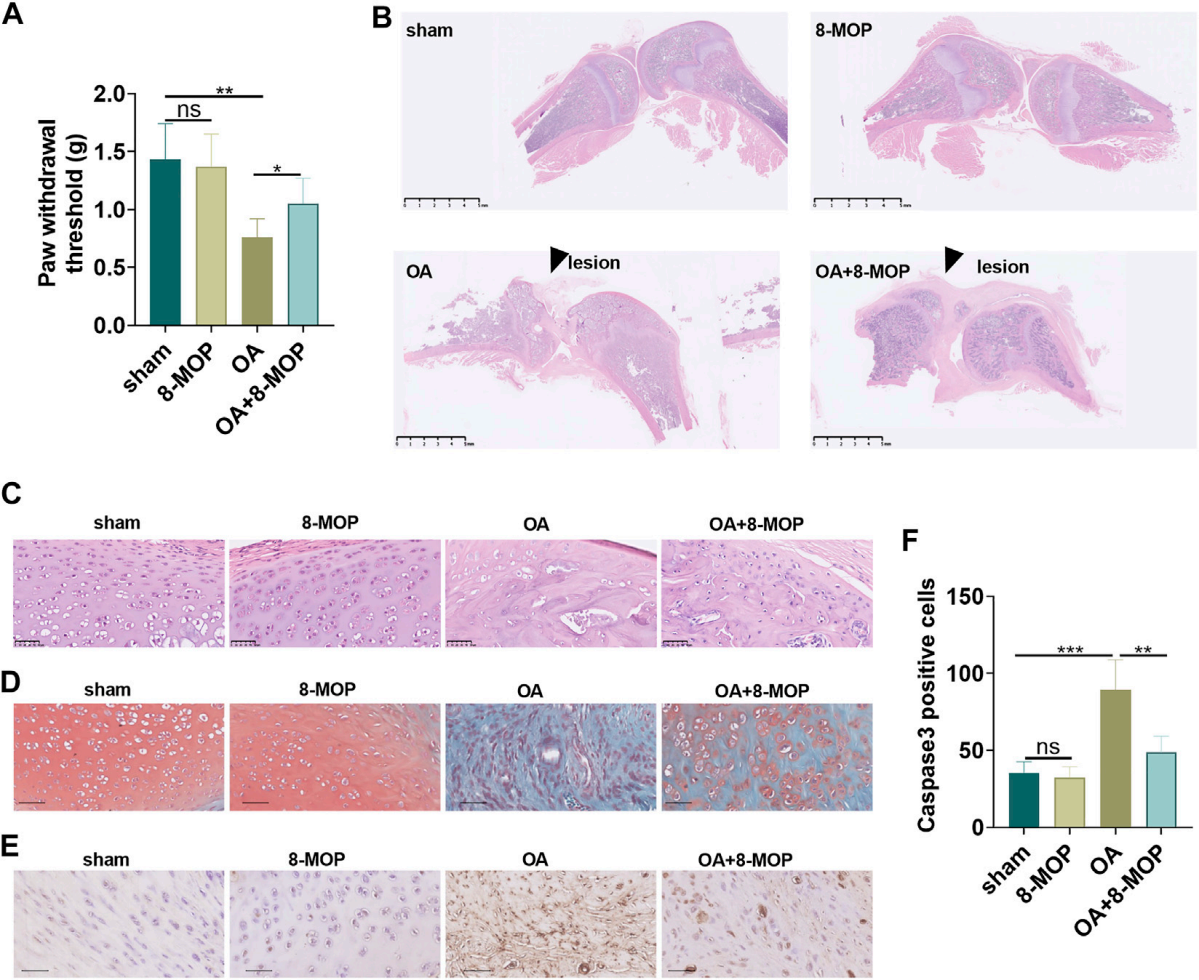
8-MOP improved the pain and articular cartilage injury in OA rats. The Wistar rat OA model was established by intraarticular injection of 4% papain. Rats were divided into four groups: The sham group, the 8-MOP (30 μmol/kg, i.p.) group, the OA group, and the OA + 8-MOP (30 μmol/kg, i.p.) group. The rats were sacrificed and the knee joint tissues were used for histopathological examination. **(A)**. The PWT was measured to assess the level of bone pain in rats. **(B–C)**. HE staining was performed for evaluating the histopathological changes of knee joint of rats. **(B)** showed the gross image of HE staining (scale bar = 5 mm), and **(C)** showed that enlarged image of the OA site (200×, scale bar = 50 μm). **(D)**. Safranin O/fast green staining was performed on the cartilage tissue sections of the knee joint of rats (200×, scale bar = 50 μm). **(E–F)**. The number of Caspase3-positive cells was determined by IHC to evaluate the level of tissue cell apoptosis (200×, scale bar = 50 μm). The apoptotic cells (labeled by Caspase3) were counted. ns *p* > 0.05, *p < 0.05, **p < 0.01, ***p < 0.001. (*n* = 5).

To probe the toxic effects of 8-MOP in rats, we established a rat OA model by intraarticular injection of 4% papain. 8-MOP was administered *via* intraperitoneal injection. The liver and kidney functions as well as skin reactions of the rats were tested or observed. HE staining showed that compared with sham or OA group, there were no significant pathological changes in liver and kidney tissues (Supplementary Figures S1A–B) after 8-MOP treatment. There was no large area of erythema or pruritus observed in the rats. Further biochemical analysis of blood showed that 8-MOP had no significant effects in affecting liver function markers (alanine aminotransferase, aspartate aminotransferas, albumin) and kidney function markers (blood urea nitrogen, total plasma protein, total bilirubin) in rats (Supplementary Figures S1C–H). The high-performance liquid chromatography-diode array detector (HPLC-DAD) results showed that after 6 h after the treatment of 8-MOP, the content of 8-MOP in the serum was 45.822 ± 1.72 μg/ml, in the osteoarthritis site of rats was 5.951 ± 0.15 μg/g tissues, indicating that 8-MOP could be delivered in to the OA site *via* intraperitoneal injection. The PWT of OA rat was determined the day after 8-MOP treatment. The findings showed that compared with the sham group, the PWT value of the 8-MOP group did not change distinctly, while the PWT value of the OA group decreased evidently. However, compared with the OA group, the PWT value of the OA + 8-MOP group was increased (*p* < 0.05, Figure 5A). HE staining, Safranin O/fast green staining and IHC were conducted to measure the cartilage sections of the rat knee joint 6 weeks after the modeling. HE staining revealed regular morphological structure of cartilage tissues in the sham and 8-MOP groups. In contrast, the articular surface was severely damaged, the number of chondrocytes decreased and the structure of cartilage tissue was significantly damaged in the OA group. However, the articular surface tended to be complete, and the cell arrangement tended to be orderly and horizontal, and chondrocytes increased in the OA + 8-MOP group (*p* < 0.05, Figures 5B,C). Safranin O/fast green staining showed no cartilage tissue damage in the sham and 8-MOP groups. In the OA group, reduced cartilage thickness and mass matrix loss were observed. The cartilage thickness of the OA + 8-MOP group seemed to be normal (*p* < 0.05, Figure 5D). The data analysis of IHC exhibited that the number of Caspase3-positive cells in the 8-MOP group was of no evident changes compared with that of the sham group, while the positive cell number in the OA group was facilitated. Compared with the OA group, the number of Caspase3-positive cells in the OA + 8-MOP group was obviously reduced (*p* < 0.05, Figures 5E,F). The above results exhibited that 8-MOP improved the pain and articular cartilage injury in OA rats.

### 8-MOP Relieved the Inflammatory and Oxidative Stress Responses in Articular Cartilage

**FIGURE 6:**
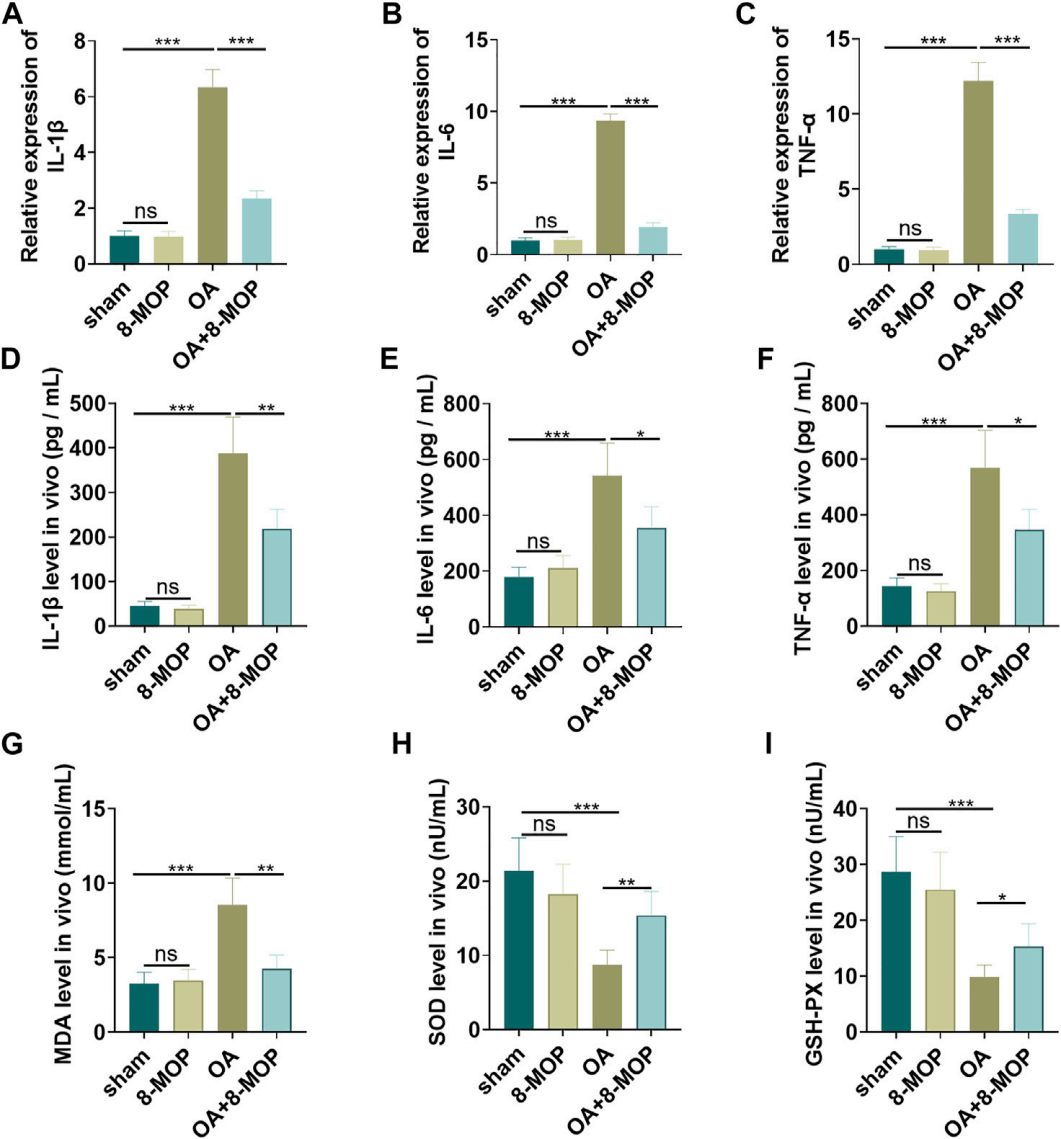
8-MOP relieved the inflammatory and oxidative stress responses in articular cartilage. The Wistar rat OA model was established by intraarticular injection of 4% papain. Rats were divided into four groups: The sham group, the 8-MOP (30 μmol/kg, i.p.) group, the OA group, and the OA + 8-MOP (30 μmol/kg, i.p.) group. **(A–F)**. The levels of inflammatory cytokines (IL-6, TNF-α, and IL-18) were detected by qRT-PCR and ELISA. **(G–I)**: The oxidative stress factors (MDA, SOD, and GSH-PX) in the OA site were measured by the MDA, SOD, and GSH-PX detection kits. ns *p* > 0.05, *p < 0.05, **p < 0.01, ***p < 0.001. (*n* = 5).

Next, the level of inflammatory and oxidative stress responses of tissues was determined by qRT-PCR and ELISA. As a result, compared with the sham group, the level of cytokines (IL-6, TNF-α, IL-18) and oxidative stress markers (MDA, SOD, GSH-PX) in the 8-MOP group was of no evident changes. Nevertheless, the levels of proinflammatory cytokines (IL-6, TNF-α, IL-18) and the membrane lipid peroxidation product MDA in the OA group were elevated, and the release of SOD and GSH-PX was dramatically reduced. However, compared with the OA group, IL-6, TNF-α, IL-18 and MDA levels were lessened while SOD and GSH-PX were increased in the OA + 8-MOP group (*p* < 0.05, Figures 6A–I). All these results indicated that 8-MOP relieved the inflammatory and oxidative stress responses in articular cartilage.

### 8-MOP Facilitated the Expression of the AMPK/SIRT1 and Inactivated NF-κB Pathway in OA Rats

**FIGURE 7:**
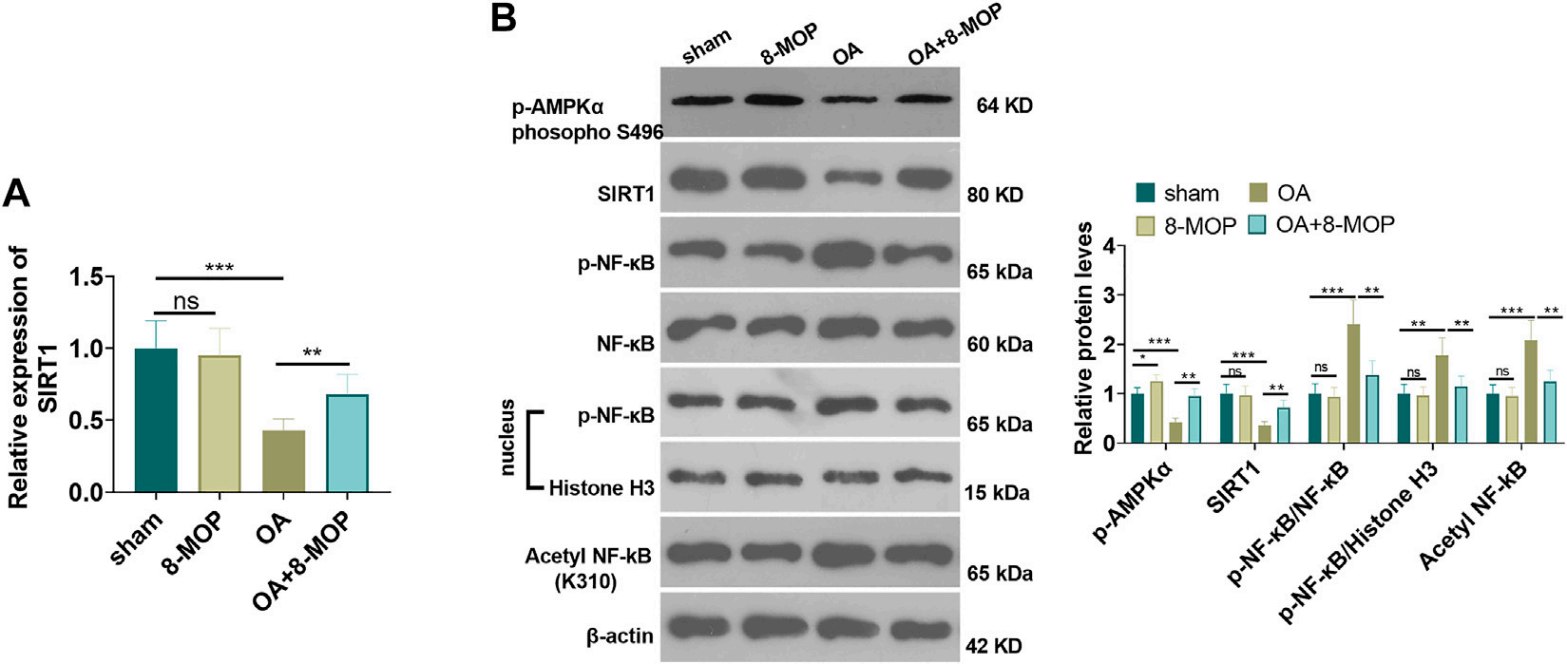
8-MOP facilitated the expression of the SIRT1/NF-κB pathway in OA rats. The Wistar rat OA model was established by intraarticular injection of 4% papain. Rats were divided into four groups: The sham group, the 8-MOP (30 μmol/kg, i.p.) group, the OA group, and the OA + 8-MOP (30 μmol/kg, i.p.) group. **(A)**. qRT-PCR was used for evaluating SIRT1 mRNA. **(B)**. The expression of *p*-AMPKα, SIRT1 and p-NF-κB p65 protein levels and acetylation NF-κB p65 was determined by Western blot. ns *p* > 0.05, *p < 0.05, **p < 0.01, ***p < 0.001. (*n* = 5).

Furthermore, the expression of SIRT1 mRNA level was tested by qRT-PCR. It was found that OA rat had lower SIRT1 mRNA in the OA site, while 8-MOP significantly enhanced its level (Figure 6A). The protein level of AMPK/SIRT1 and NF-κB was detected by Western blot. The results revealed that compared with the sham group, the expression of SIRT1 and NF-κB did not change obviously in the 8-MOP group. However, p-AMPKαand SIRT1 were both inhibited in the OA site, and expression of p-NF-κB p65 in the whole cells and nuclear as well as acetylated NF-κB p65 were all promoted (compared with sham group). Under the treatment of 8-MOP, the expression of p-AMPKαand SIRT1 was enhanced and the expression of p-NF-κB p65 in the whole cells and nuclear as well as acetylated NF-κB p65 was all reduced in the OA + 8-MOP group (*p* < 0.05, Figure 7B). These results showed that 8-MOP facilitated the AMPK/SIRT1 and downregulated NF-κB pathway expression in OA rats.

### Inhibiting SIRT1 Attenuated the Protective Effect of 8-MOP on Osteoarthritis Rats

**FIGURE 8:**
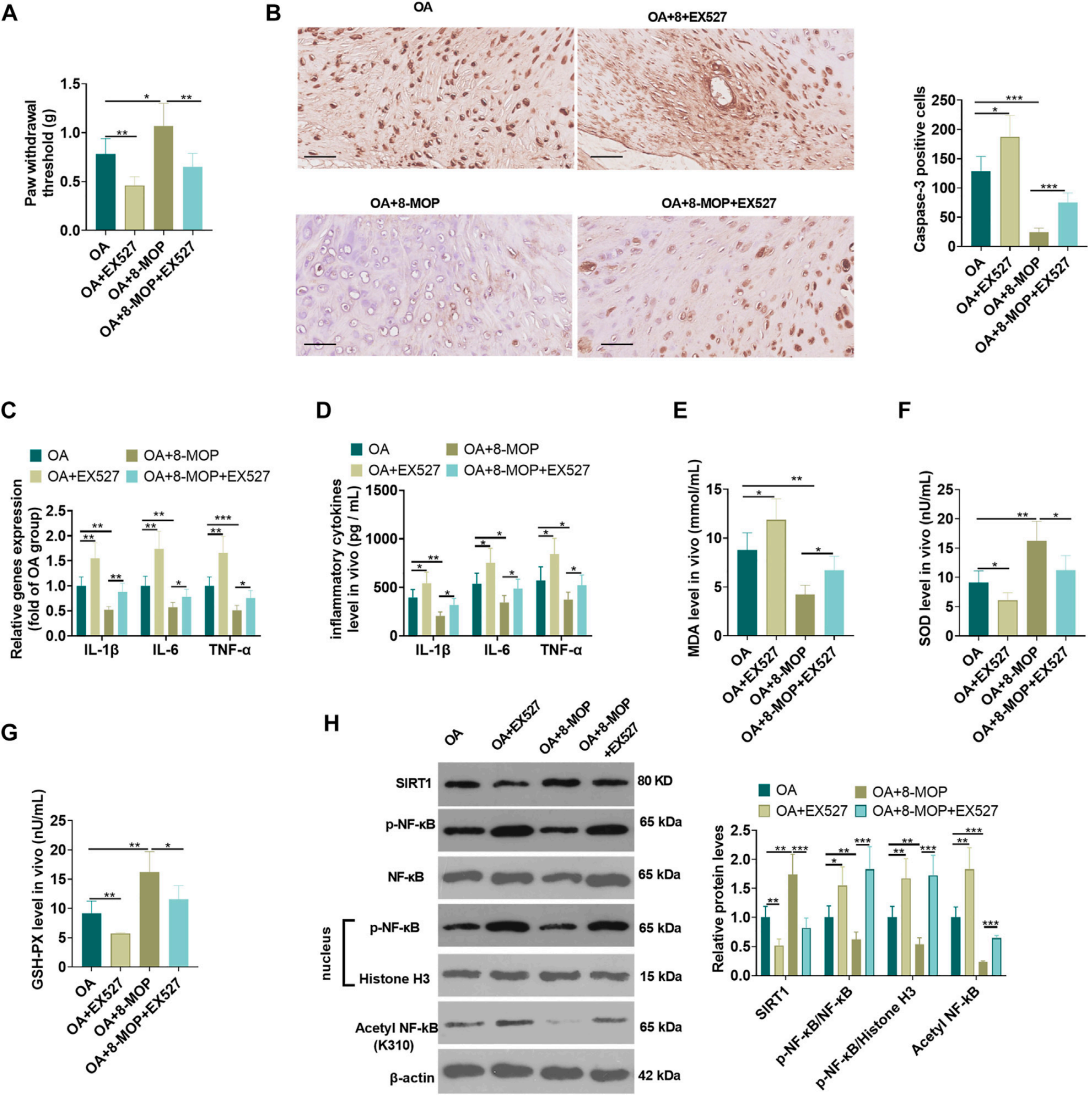
Inhibition of SIRT1 attenuated the protective effect of 8-MOP on OA rats. The Wistar rat OA model was established by intraarticular injection of 4% papain. Rats were divided into four groups: The OA group, the OA + EX527 (20 μmol/kg body weight) group, the OA + 8-MOP (30 μmol/kg, i.p.) group, the OA + EX527 (20 μmol/kg body weight) +8-MOP (30 μmol/kg, i.p.) group. **(A)**. The PWT was measured to assess the level of bone pain in rats. **(B)**. Caspase3-positive cells were counted by IHC. **(C–D)**. The levels of inflammatory cytokines (IL-6, TNF-α, and IL-18) were detected by qRT-PCR and ELISA. **(E–G)**: The oxidative stress factors (MDA, SOD, and GSH-PX) in the OA site were measured by the MDA, SOD, and GSH-PX detection kits. **(H)**. The expression of SIRT1, p-NF-κB p65 protein levels and acetylation NF-κB p65 was measured by qRT-PCR and Western blot, respectively. *p < 0.05, **p < 0.01, ***p < 0.001. (*n* = 5).

On the basis of the establishment of a rat OA model, 8-MOP and/or the SIRT1 inhibitor EX527 were used for dealing with the OA rats. The analysis of pain threshold detection showed that compared with the OA or OA + 8-MOP group, EX527 reduced the pain threshold of rats (*p* < 0.05, Figure 8A). The results of IHC revealed that compared with the OA or OA + 8-MOP group, the number of Caspase3-positive cells was increased in both the OA + EX527 and OA + 8-MOP + EX527 group (*p* < 0.05, Figure 8B). qRT-PCR and ELISA analysis showed that, in comparison with the OA or OA + 8-MOP group, EX527 promoted the expression of inflammatory cytokines (including IL-1β, IL-6, TNF-α) (*p* < 0.05, Figures 8C,D). The oxidative stress markers in the OS site were examined. It was found that after the treatment with EX527, the MDA level was increased, while the levels of SOD and GSH-PX were impeded (vs. OA or OA + 8-MOP group, *p* < 0.05, Figures 8E–G). We also detected SIRT1 and NF-κB in the OA site by western blot. The results suggested that EX527 notably inhibited the expression of SIRT1 and promoted the expression of p-NF-κB p65, acetylated NF-κB p65 (vs. the OA or OA + 8-MOP group) (*p* < 0.05, Figure 8H). All the findings indicated that inhibition of SIRT1 attenuated the protective effect of 8-MOP on OA rats.

## Discussion

**FIGURE 9:**
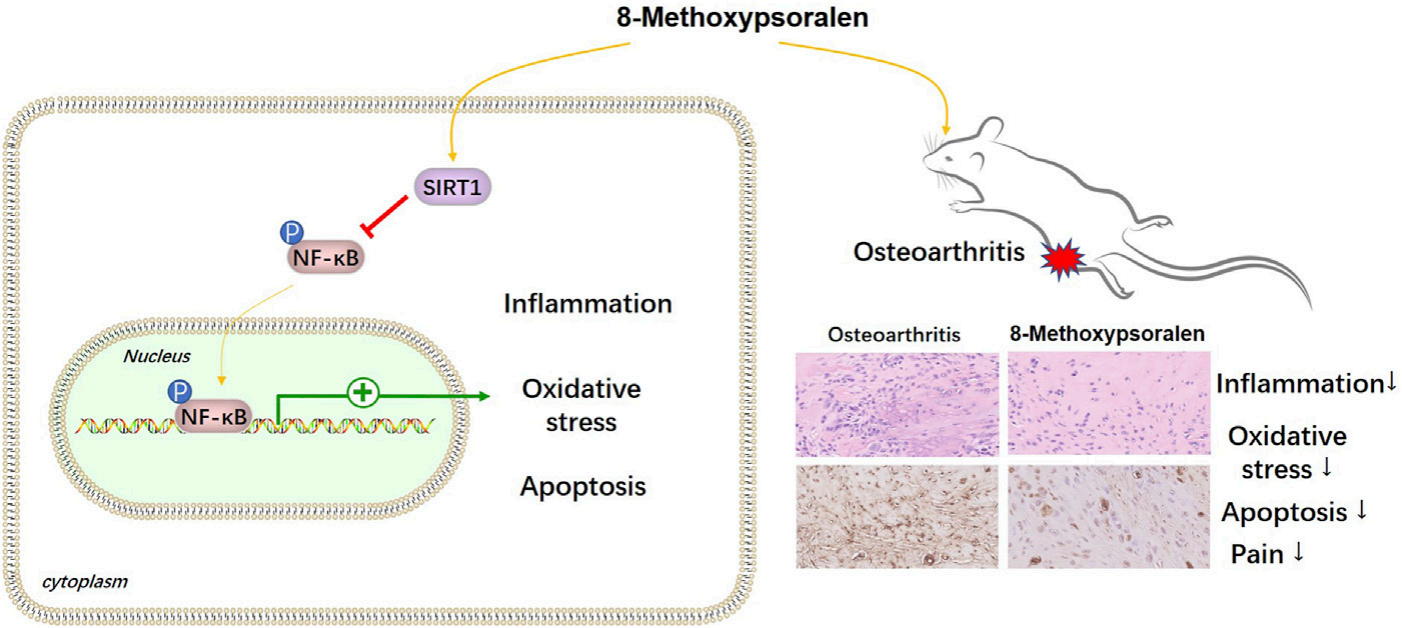
The mechanism diagram. 8- MOP alleviates pain, inflammatory and oxidative stress responses in OA rats through the upregulation SIRT1 and inactivation of NF-κB pathway.

OA is the most prevalent form of arthritis (Xia et al., 2014), and the current therapies still have many limitations, such as high recurrence rate and heavy economic burden, etc., Therefore, there is an urgent need for a safe and effective drug with molecular targeting characteristics to ease OA. Fortunately, in this study, we found that 8-MOP inhibited inflammatory and oxidative stress responses *in vitro* and *in vivo* through the SIRT1/NF-κB pathway (Figure 9).

OA is accompanied with complex pathological change. In addition to the dysfunction of chondrocytes and synovial cells, immune cell infiltration is also a main characteristic of OA (de Lange-Brokaar et al., 2012). Recently, increasing studies have revealed that the inflammatory response caused by macrophages affects the progression of OA (Lu et al., 2021). Generally, macrophages are activated and differentiate into pro-inflammatory M1 type or anti-inflammatory M2 type under different stimulation (Sun et al., 2020). Studies have shown that macrophage polarization is considered to be the key node of OA inflammatory response (Xie et al., 2019). For example, Zhang h et al., Found that M1 polarization of synovial macrophages aggravates collagenase induced osteoarthritis by promoting the secretion of r-spondin-2 (Zhang et al., 2018). IL-1β is a key mediator of cellular inflammatory responses (Lopez-Castejon and Brough, 2011). A recent study has suggested that osteoarthritic chondrocytes stimulate inflammasome activation and increase mature IL-1β production in macrophages (Ni et al., 2019). Multiple studies have applied IL-1β to induce the *in vitro* OA model of chondrocytes (Kato et al., 2014; Cai et al., 2019).

In this study, we stimulated rat chondrocytes with IL-1β (20 ng/ml) for 24 h to induce the *in vitro* OA model. Our study revealed that IL-1β suppressed the proliferation of chondrocytes and promoted their apoptosis, and promoted cellular inflammatory and oxidative stress responses. These experimental findings indicated that IL-1β induced the *in vitro* OA model. On the basis of the cell experiment, we carried out an animal experiment. A rat OA model was induced by intraarticular injection of 4% papain (Şükür et al., 2016; Cheng et al., 2019). Our results showed that compared with the sham group, injection of papain distinctly facilitated the matrix degradation and bone pain threshold of cartilage tissues of the rat knee joint and promoted the release of inflammatory cytokines and oxidative stress factors as well. These results indicated that 4% papain induced the rat OA model.

Based on the establishment of *in vitro* and *in vivo* OA models, 8-MOP treatment was utilized to explore its role in OA. It was reported that 8-MOP was originally a compound for treating skin diseases and ultraviolet radiation. Some subsequent researches have indicated that 8-MOP also has distinct anti-tumor, anti-inflammatory, and antioxidant activities (Nicolis et al., 2009; Liu et al., 2012; de Oliveira et al., 2016). For example, 8-MOP has been confirmed to protect bovine mammary epithelial cells from lipopolysaccharides-induced inflammatory damage by inhibiting the JAK/STAT and NF-κB pathways (Li et al., 2019). In this study, it was found that 8-MOP hampered cell apoptosis and promoted cell proliferation in chondrocytes compared with that of IL-1β treatment alone. Additionally, 8-MOP alleviated cartilage damage and bone pain in OA rats. More importantly, 8-MOP inhibited the inflammatory and oxidative stress responses in the OA model both *in vivo* and *in vitro*. All these experimental results verified the inhibitory role of 8-MOP in OA.

As a member of Sirtuins, which are widely involved in multiple cellular functions related to metabolism, cell cycle, aging, inflammation, apoptosis, cell proliferation, and DNA repair (Vachharajani et al., 2016), SIRT1 has been recognized as a vital regulator in OA (Matsushita et al., 2013). For instance, lncRNA LUADT1 elevates the SIRT1 expression by targeting miR-34a, thereby inhibiting the apoptotic rate of chondrocytes (Ni et al., 2020). Another study identified that curcumin suppresses the PERK-eIF2α-CHOP pathway by facilitating the expression of SIRT1, thereby inhibiting the oxidative stress of rat chondrocytes and OA progression in rats (Feng et al., 2019b). These studies indicated that relevant drugs or genes could play an inhibitory role in OA by upregulating the expression of SIRT1. NF-κB pathway activation is a crucial factor of OA development via modulation inflammation and apoptosis (Lepetsos et al., 2019). Targeting NF-κB pathway was considered to be an important pathway for controlling the development of chondrocytes and pathological injury of cartilage (Jimi et al., 2019). One study revealed that inhibition of G protein-coupled receptor kinase 5 (GRK5) attenuated cartilage degradation by reducing NF-κB-mediated catabolic responses, thereby relieving OA progression in mice (Sueishi et al., 2020). In addition, Protectin DX has been verified to inhibit the NF-κB pathway by promoting AMPK, and thus alleviating IL-1β-induced chondrocyte inflammation and inhibiting OA progression in rats (Piao et al., 2020). Interestingly, 8-Methoxypsoralen (also known as Xanthotoxin) has been identified to repress NF-κB activation and inflammatory reactions (Lee et al., 2017; Li et al., 2019; Hindam et al., 2020). In this study, we found that 8-MOP obviously promoted the expression of SIRT1 and inhibited the expression of NF-κB in the OA model. Further mechanism researches identified that EX527 (SIRT1-specific inhibitor) attenuated the inhibitory role of 8-MOP in OA. This indicated that the inhibition of 8-MOP on OA was at least partially mediated by promoting SIRT1 and thus inhibiting the NF-κB pathway.

Interestingly, we found that 8-MOP enhanced *p*-AMPKα level in the chondrocytes and cartilage tissue, which is consistent with the upregulation of SIRT1. Several studies have confirmed that activation of AMPK pathway exerts anti-inflammatory in OA and relieves the apoptosis of chondrocytes, which is partly dependent on repressing NF-κB pathway-mediated inflammatory reactions (Yang et al., 2019; Zhang et al., 2020). AMPK usually functions as a cellular energy sensor and can activate a number of key metabolic enzymes through phosphorylation regulation (Garcia and Shaw, 2017). Interestingly, enhancing AMPK in OA could also promote SIRT1 expression, thereby inhibiting NF-κB pathway activation (Liu et al., 2020; Wang et al., 2020). The above studies suggest that the AMPK/SIRT1/NF-κB pathway plays a crucial role in OA, and our prelim study suggested that 8-MOP relieves OA progression potentially through activating AMPK/SIRT1 axis.

In summary, this study found that 8-MOP played an anti-inflammatory and anti-oxidative role through the SIRT1/NF-κB pathway and suppressed apoptosis and anti-proliferation of chondrocytes, thereby inhibiting OA progression in rats. The activation of AMPK pathway seems to be involved in 8-MOP-mediated effects in OA. This provides a new approach and research direction for OA treatment. However, several weaknesses are still existed. On the one hand, a wider range of doses of 8-MOP should be administered into the rats to confirm the toxic effects of 8-MOP. On the other hand, the specific mechanism of AMPK/SIRT1 upregulation by 8-MOP needs further exploration. 8-MOP probably upregulates AMPK or SIRT1 *via* promoting their DNA transcription, targeting their upstream or the cellular membrane receptors, inducing acetylation and methylation of AMPK/SIRT1 axis, or by the other ways.

## Data Availability

The datasets presented in this study can be found in online repositories. The names of the repository/repositories and accession number(s) can be found in the article/Supplementary Material.
