# Supplementary figures and images for: 8-Methoxypsoralen has Anti-inflammatory and Antioxidant Roles in Osteoarthritis Through SIRT1/NF-κB Pathway
Source: Front Pharmacol. 2021 Sep 6;12:692424. doi: 10.3389/fphar.2021.692424 (PMC8450503; doi:10.3389/fphar.2021.692424)

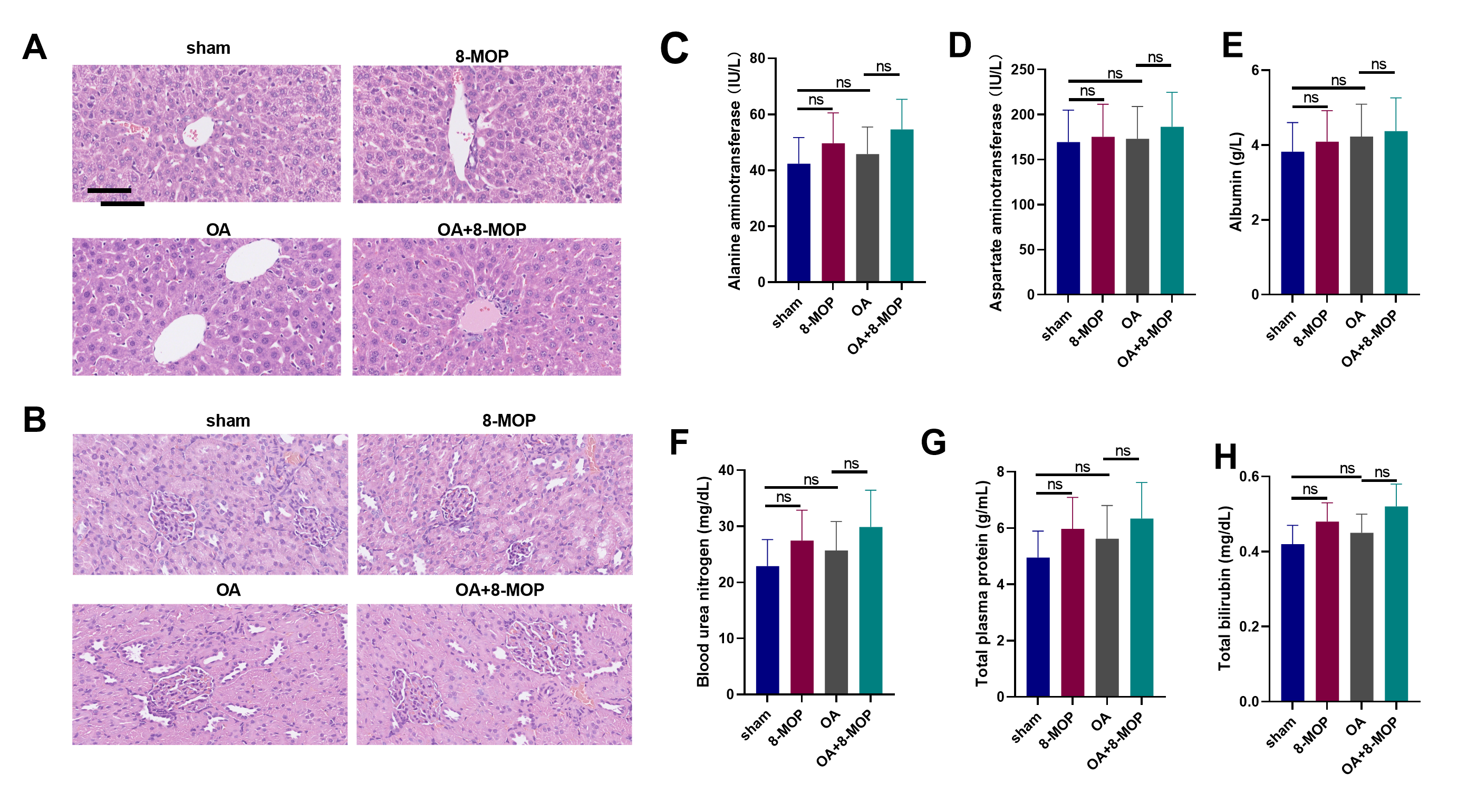

Supplement: Supplementary file 1 [file Image1.TIF]
